# Atherogenic Plasma Index or Non-High-Density Lipoproteins as Markers Best Reflecting Age-Related High Concentrations of Small Dense Low-Density Lipoproteins

**DOI:** 10.3390/ijms23095089

**Published:** 2022-05-03

**Authors:** Sylwia Płaczkowska, Katarzyna Sołkiewicz, Iwona Bednarz-Misa, Ewa Maria Kratz

**Affiliations:** 1 Department of Laboratory Diagnostics, Teaching and Research Diagnostic Laboratory, Wroclaw Medical University, Borowska 211a St., 50-556 Wroclaw, Poland; 2 Department of Laboratory Diagnostics, Division of Laboratory Diagnostics, Wroclaw Medical University, Borowska 211a St., 50-556 Wroclaw, Poland; katarzyna.solkiewicz@umw.edu.pl; 3 Department of Biochemistry and Immunochemistry, Division of Medical Biochemistry, Wroclaw Medical University, M. Skłodowskiej-Curie 48/50 St., 50-369 Wroclaw, Poland; iwona.bednarz-misa@umw.edu.pl

**Keywords:** high-density lipoproteins (HDL), low-density lipoproteins (LDL), small dense low-density lipoproteins (sdLDL), cardiovascular disease (CVD), young adults, atherogenic indexes

## Abstract

The study aimed to assess the strength of the relationships between small dense low-density lipoproteins (sdLDL) and other parameters describing metabolic disorders and determine which of the lipid profile parameters can be used as markers of increased sdLDL concentration. The proposed model of sdLDL (examined by heparin–magnesium precipitation method) as a function of lipid parameters and atherogenic plasma indexes non-high-dense lipoproteins (non-HDL) and total cholesterol to high-dense lipoprotein ratio (TC/HDL), Atherogenic plasma index (API) is based on data from 485 participants divided into two age groups, <35≥ years. In multiple linear regression, sdLDL concentration was associated with the concentration of non-HDL-C (*p* = 0.043) and API value (*p* < 0.001) in participants <35 years, and with non-HDL-C (*p* < 0.001) and triglycerides (*p* = 0.020) concentration ≥35 years. The presence of abnormal values of API in participants <35 years and non-HDL-C in participants ≥35 years is a significant factor increasing the chances of the highest sdLDL (≥1.03 mmol/L) corresponding to Q4 in people without metabolic disorders. Different lipid parameters and atherogenicity indexes are associated with a high concentration of sdLDL depending on the age group. Abnormal API <35 years and non-HDL ≥35 years are associated with the highest sdLDL values and may be an indication for further specialist diagnosis of cardiovascular disease risk factors.

## 1. Introduction

Cardiovascular diseases (CVD) are the leading cause of death worldwide. The lipid composition of circulating blood is particularly important in the development of this group of diseases. Among the routinely determined laboratory lipid parameters, LDL cholesterol (LDL-C) is of particular importance, currently recognized as a target parameter in the primary and secondary prevention of cardiovascular events [1,2]. However, LDL-C is a heterogeneous group of molecules that differ significantly in size (diameter 20–25 nm) and density (d) from 1.019 to 1.063 g/mL [1,3]. LDL subclasses are distinguished on the basis of their density (differing in terms of their physicochemical, metabolic, and functional properties as well as their ability to participate in atherogenesis) [1]. The differentiation of the atherogenicity of LDL subclasses results from the different lipid and protein compositions, plasma residence time, an affinity for cellular LDL receptors and arterial wall proteoglycans, and susceptibility to oxidative modifications [3]. Many studies have been conducted on LDL subclasses, particularly small dense LDL (sdLDL) (d = 1.044–1.063 g/mL) [3,4,5]. According to the available evidence, the small dense subclass of LDL is the most atherogenic component of blood [4], and the determination of its concentration can be considered the perfect alternative to LDL-C measurements [6]. The significant atherogenicity of sdLDL molecules is conditioned by several factors. Their small size enables them to penetrate easily into the arterial wall, while their high affinity for proteoglycans in the arterial wall leads to prolonged residency in the subendothelial space. On the other hand, the clearance of sdLDL from blood plasma is delayed as a result of the reduced affinity of sdLDL to LDL receptors in comparison to larger LDL-C particles. Small, dense LDL particles are also highly susceptible to oxidization [7]. All the features mentioned above explain the increase in expression of the atherogenic small, dense LDL subclass observed in many metabolic disorders. Excessive formation of sdLDL molecules is associated with numerous genetic and environmental factors, and the direct cause of their production in circulation is abnormal lipoprotein metabolism [3]. One of the non-modifiable risk factors for cardiovascular diseases is age, which significantly alters the lipid profile to a more atherogenic one [8,9,10]. The atherogenicity of the above-mentioned sdLDL particles is best documented in middle-aged people, but the development of cardiovascular disease starts early and the first symptoms and disorders appear already in adolescence. Cardiovascular prevention screening programs are rarely administered to people under 35-40 years of age [11] because these types of projects are mainly based on the routine 10-year CVD risk assessment cards for people over 40 years old [12]. However, the metabolic disorders considered to be a significant risk factor for cardiovascular diseases are already found in the youngest group of adults [10] and do not result only from genetic factors [3]. This is due to the increasing number of risk factors for cardiovascular diseases such as obesity, insufficient physical inactivity, and poor diet among young adults in highly developed countries in the last two decades [13]. This implies a situation where a low absolute CVD risk in young people may mask significant relative risk connected with the occurrence of a single strong risk factor like a lipids disorder and which could be modified thanks to early therapeutic intervention. At the same time, studies are conducted to assess the metabolic disorders in early adulthood as independent risk factors for cardiovascular diseases in later years [14,15]. Therefore, it seems justified to conduct research on the epidemiology and importance of different CVD risk factors also among young adults from the age of 18.

In clinical practice, in addition to the most commonly measured LDL-C concentration, several atherogenicity indicators are known to predict the likelihood of cardiovascular events or to be used as target values for treatment [16,17,18]. Some of them, such as the frequently reported ratio of total cholesterol (TC) to high-density lipoprotein cholesterol (HDL-C) (TC/HDL) [16] and the most recently extensively studied atherogenic plasma index (API) [19,20], can be calculated based on lipid parameters determined in routine laboratory practice. Since both indicators are calculated as a proportion between concentrations of cholesterol fractions, they illustrate not only the level of a given parameter but also their mutual proportions. It is worth noting that disturbances in the proportion of lipoprotein fractions, particularly HDL-C and triglycerides (TG), are the main factors leading to the formation of sdLDL.

In the study, we hypothesize that the association of sdLDL concentration with levels of traditional laboratory CVD risk factors and atherogenicity indexes is different for the younger population, due to their different blood lipid profile and the shorter duration of adverse blood composition. We have also assumed that it is possible to indicate a sensitive and specific lipid parameter and/or an atherogenicity index that will indicate the group of people with the highest probability of high levels of sdLDL and that the concentration of this parameter may depend on the patient’s age. Therefore, the purpose of our research was to answer the following questions: (i) what relationships exist between sdLDL levels and expression of other parameters describing glucose (GLU), basic lipid profile (TC, HDL-C, non-HDL, LDL-C, TG), and atherogenicity indexes (API, TC/HDL), and how strong are they in groups of <35 and ≥35 year-old participants from the same population? and (ii) is it possible that the expression of parameters mentioned above can be used as markers related to increased sdLDL concentration and to select a group of people, particularly at risk of cardiovascular disease development in the future?

## 2. Results

Characteristics of all study participants in age groups <35 and ≥35 years are shown in Table 1.

In both age groups over 60% of participants were women. Significantly higher values were found for the age group ≥35 years only in glucose, total cholesterol, LDL-C, non-HDL, triglycerides, and sdLDL concentrations. For the parameters analyzed when taking into account the sex of the participants, significantly higher values were observed for TC/HDL for both sexes and API only for older women.

The association of sdLDL concentration with metabolic parameters and atherogenic indexes was investigated using a Pearson correlation analysis in groups distinguished by age (data were logarithmically transformed when appropriate) and are presented in Table 2. 

Small, dense LDL correlates with blood lipid parameters, but with different powers. For the younger age group, the highest but weak-to-moderate correlation coefficient was observed for API, TG, and TC/HDL. For the older group, moderate correlation power was revealed for TC, LDL-C, non-HDL, and TG, however, there was no significant relationship between sdLDL and HDL in this age group. Glucose showed no significant correlation with sdLDL in either age group. It was also shown that values of correlation coefficient were generally higher for the group of older participants however, these differences were significant only for TC, HDL-C, LDL-C, and non-HDL-C. 

In the next step of the correlation analysis, we investigated which of the lipid parameters and atherogenicity indicators could be independent predictors of sdLDL, by multiple regression analysis. The parameters whose correlation coefficients were significant and higher than 0.2 were selected as independent predictors. At the same time, only non-HDL-C was taken into account, because total cholesterol, LDL-C, and non-HDL-C are highly correlated (*r* > 0.9), meaning that they contribute the same amount of information to the multivariate regression model. The results of this analysis, presented in Table 3, indicate that in the group of younger people API and non-HDL cholesterol are independent positive predictors of sdLDL concentration (R^2^ for model = 0.121), while in the group of older subjects the same features are assigned to TG and non-HDL (R^2^ for model = 0.216). This means that the concentration of sdLDL significantly increases with the increase of non-HDL concentration in the analyzed age groups. Additionally, in the younger age group, sdLDL is also related to API, i.e., the TG to HDL-C ratio, while in the older age group only TG concentration remains significantly related to the concentration of sdLDL.

We also determined sdLDL quartiles in a group of healthy people (*N* = 152), including participants who showed no disturbances in the analyzed metabolic parameters: glucose, TC, HDL-C, LDL-C, and TG. The characteristics and comparison of the studied parameters in healthy subjects regarding sdLDL quartiles are presented in Supplementary Table S1, and there were no differences for any of the studied parameters according to sdLDL quartiles among healthy participants. Thanks to the separation of this group, we determined the values characteristic for the studied population of healthy people, which were used for further comparisons and assessment of differences in the lipid profile of the analyzed age groups.

In the next step of the analysis, the studied parameters were compared in all study participants, divided according to quartiles of sdLDL determined for the group of healthy subjects. The results of this analysis are shown in Supplementary Materials Table S2. 

**Figure 1:**
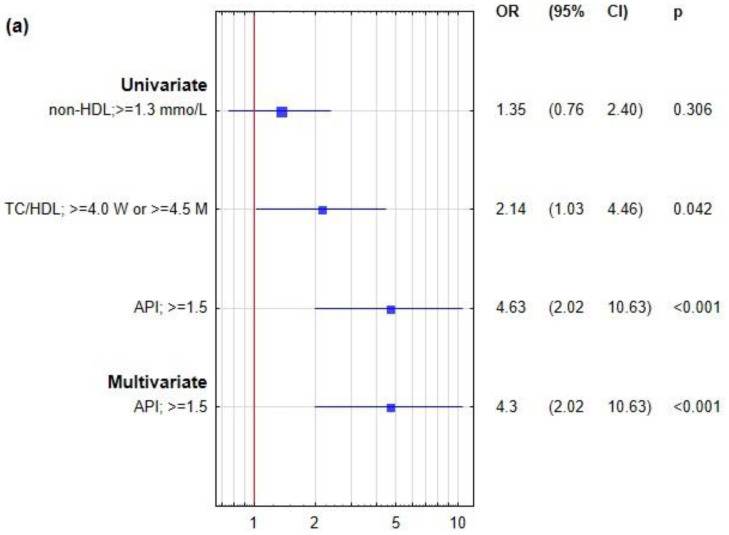

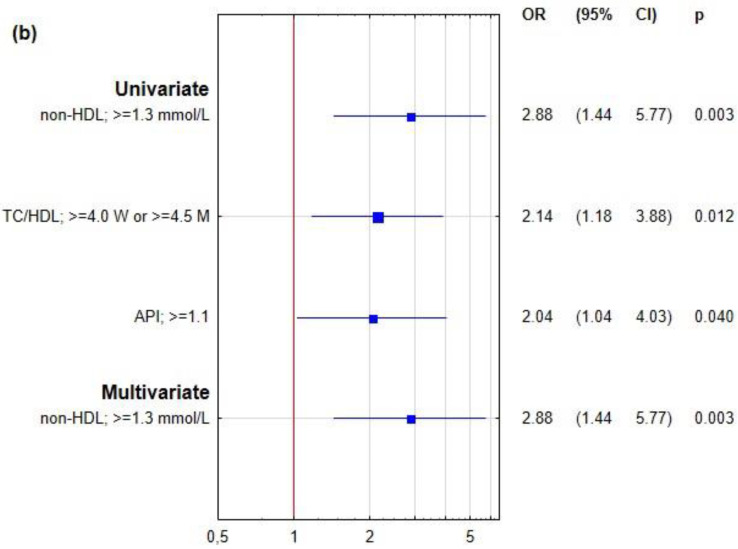
Univariate and multivariate analysis of logistic regression was performed for the Upper Quartile of sdLDL in age groups <35 years (Panel **a**) and ≥35 years (Panel **b**). The multivariable model included sex, non-HDL-C, TC/HDL, and API, provided as dichotomous variables divided according to the criteria described in the Material and Methods section. Variables selection was conducted by the forward stepwise procedure. Non-HDL—non-high-density lipoprotein cholesterol; API—atherogenic plasma index; RC—regression coefficient; OR—odds ratio; CI—confidence interval; W—women; M—men. A *p*-value < 0.05 was considered significant.

The division of atherogenic indexes was made based on the cut-offs described in the Materials and Methods section. From two strongly correlated parameters, LDL and non-HDL, the latter was chosen, because it is indicated in up-to-date literature as preferable to LDL. The frequency of specific metabolic abnormalities in the studied age groups is presented in Supplementary Table S3. In Figure 1, univariate analyses showed the significant association of sdLDL Upper Quartile with API and TC/HDL in both age groups, while abnormal non-HDL-C was revealed as a significant predictor of Q4 sdLDL only for the older group. 

The results showed that for young subjects with abnormal values of TC/HDL and/or API, the hazard of sdLDL > 1.03 mmol/L (Q4) increased from 2.14 to 4.63 in comparison to subjects with normal values of these parameters. For the age group ≥35 years, a similar association was observed for all three of the studied indexes: non-HDL, TC/HDL, and API. Moreover, odds ratios for these parameters were higher in the younger age group, especially for API (nearly twice as high). Stepwise logistic regression conducted in the next step revealed that upon taking into account the studied atherogenic indexes concerning sex, API level remained an independent predictor of sdLDL concentration corresponding to Q4 for the younger group only. A different situation was observed in the older age group, where only abnormal non-HDL was revealed to be an independent predictor of Q4 sdLDL. 

## 3. Discussion

Our findings indicate that subjects with identified specific metabolic disorders, in age groups both below 35 and ≥35 years, tend to have a significantly higher risk of sdLDL concentration in the upper quartile. Moreover, the association of sdLDL level with concentrations of standard lipid profile parameters and atherogenic indexes differs in the distinguished age groups. The highest levels of sdLDL are also disclosed in young adults who participated in our study. 

The sdLDL has been described as a new potential marker of cardiovascular risk quite extensively in the scientific literature, but mainly in older age groups than those investigated in our study, or among people with specific metabolic disorders [5,21,22,23,24]. The trend toward deteriorating values of classic metabolic disorder parameters with age, observed in this study, is similar to the results obtained by other authors [10,25,26]. However, in our study, participants of both sexes and age groups showed similar HDL concentrations. These observations are comparable to the results obtained for the Polish population by Buciński et al. [27]. The authors indicated the highest age-dependent differences for levels of triglycerides, while the average level of HDL did not change significantly across the age range of 35–55 in men or women [27]. Higher values in older participants were also observed in our study for sdLDL, whose concentration is related to decreased levels of HDL and hypertriglyceridemia [21,28]. In their large cross-sectional study, Izimida et al. [29] observed an age-related trend for the increase of sdLDL levels in the Japanese population among participants over the age of 40. Similarly, Kikkawa et al. [30] reported a trend towards an increase in sdLDL concentrations with age for the middle-aged Japanese population, and higher concentrations in men across all the studied 10-year intervals. Nevertheless, Fernández-Cidón et al. [31] documented that for the Mediterranean population aged ≥19 and <60 years, there were no differences in sdLDL levels between the 10-year intervals in the distinguished age groups. However, the latter study was conducted in a group with an extremely small number of participants (*n* = 45), therefore, the results obtained by the authors are uncertain. Considering the data presented by the above-mentioned authors, it is reasonable to take into account the age of participants when analyzing results obtained for a wide age range, as was the case in our study. 

Besides the fact that the concentration of most lipid parameters differed in the analyzed age groups, they also showed different strengths of correlation with sdLDL concentrations, depending on the age of participants. It has been indicated that an increase in sdLDL concentrations is strongly associated with an increase in triglycerides and a decrease in HDL-C concentrations [32,33]. In our study, we also confirmed significant correlations, similar in power, between sdLDL and TG levels, while for HDL-C concentrations the obtained results do not indicate such significant correlations as those described by other authors [34,35]. A very weak inverse relationship between sdLDL and HDL levels has been demonstrated only for young adults. The highest correlations in the group of people ≥35 years were found for concentrations of total and non-HDL cholesterol. Generally, a stronger association of sdLDL with lipid parameters was observed in older age, which may be attributed to a longer duration of metabolic disorders. The strength and direction of these relationships observed in middle-aged and older adults differ in various studies [34,36,37], but generally, they are similar to the results obtained by us. Among the analyzed lipid parameters and atherogenicity indexes, regardless of age, the independent and common predictor of sdLDL concentration was non-HDL-C. As non-HDL-C is an integrated complex of all lipoprotein particles containing apolipoprotein B, that is LDL, VLDL, IDL, chylomicrons, remnants, and lipoprotein (a), it is considered to be a very good marker, especially in predicting cardiovascular events. Importantly, it can be calculated directly from the values of routine lipid profile at no extra cost [38], measured in both fasting and the post-prandial state, and is valid for very high concentrations of TG (>4.5 mmol/L) [39]. Similar to our analysis, the multiple regression of lipid factors and glucose was performed by Srisawasdi et al. [34] for patients of the age corresponding to our older group of participants. The results are similar to ours, indicating non-HDL as the strongest (next to LDL) predictor of sdLDL concentration. However, the correlation coefficients were much higher than those observed in our study, which may result from the use of other methods of sdLDL determination (the Denka homogeneous method). In contrast to the study by Srisawasdi et al. [34], in addition to non-HDL, we also showed an independent and significant positive association with sdLDL for API in young adults and TG in older adults. According to previously published data by other authors, this indicates the fact that the concentration of sdLDL is modified by TG level, but such a direct relationship is only visible in people >35 years of age. However, in the younger age group, this dependence is additionally modified by HDL concentration, because API is calculated as the ratio of TG concentration to HDL level. This shows that the increase in sdLDL level in young people is dependent on TG concentration in a situation in which HDL reduction occurs simultaneously.

In the study, we also searched for answers to the following questions: which of the studied atherogenic indexes—non-HDL, API, and/or TC/HDL—is associated with an increase in sdLDL concentration, and are such relationships similar in both analyzed age groups? Finding the answers to the above questions will enable us to select a parameter most likely to indicate people who require further in-depth diagnostics due to the high concentration of very atherogenic sdLDL particles. Given the lack of cut-off values for sdLDL values above which increased CVD risk can be identified, we have used Quartile 4 (sdLDL ≥1.03 mmol/L) as a concentration indicative of significant risk. Our results revealed that the strongest predictor of the occurrence of the highest sdLDL concentrations in young people is abnormal API value, which increases the chance of sdLDL concentration above 1.03 mmol/L almost fivefold. In the ≥35 year group, the same applies mainly to the abnormal value of non-HDL, which increases the chance of the highest sdLDL values threefold. The presence of abnormal values of all biochemical markers of atherogenicity should be an indication for taking appropriate preventive measures. However, concerning sdLDL, API is particularly important in people <35 years of age, while an increased value of non-HDL is of more significance in the group ≥35 years, for whom further diagnostics, such as direct determination of the concentration of sdLDL, are recommended.

## 4. Materials and Methods

### 4.1. Study Design

In this cross-sectional study, the participants were volunteers who obtained information about it from social media (Facebook, Instagram,) and students of the University of the Third Age at the Medical University of Wroclaw. The criteria for inclusion were: age over 18 years, willingness to participate in the study, and no contraindications to venous blood collection. The exclusion criteria were: a history of diabetes, liver or kidney failure, past cancer, acute infections during the 2 weeks preceding the study, taking lipid-lowering or anti-diabetes medications during the 3 months preceding the study, or taking anti-allergic drugs during the 3 months preceding the study, as reported by the participants. Recruitment for the study was carried out in 2019–2020. The study procedures followed in the study were conducted in agreement with the Helsinki II declaration and the protocol was approved by the Bioethics Human Research Committee of the Wroclaw Medical University (Permission No. KB-86/2020). All persons included gave their informed consent in writing, after being informed about the purpose and procedure of the study.

### 4.2. Participants and Blood Sample Collection

Out of almost 550 people who responded to our invitation, 485 volunteers were finally enrolled in the study, 64.5% of whom were women. The lower reportability of men resulted from their lower degree of willingness to participate in the project, mainly due to the fear of peripheral blood donation. Volunteers were admitted to the Teaching and Research Diagnostic Laboratory, Department of Laboratory Diagnostics, Wroclaw Medical University, between 7.00 and 9.00 a.m., after a minimum 8 h fast. Peripheral blood samples were collected into two test tubes to obtain plasma and serum samples. Plasma was separated from the whole blood collected onto EDTA within 30 min, according to standard procedure. Whole blood for serum preparation was collected into tubes containing a clot activator and left to clot for 30 min at room temperature. Then the serum was separated from the clot according to standard procedure. Aliquots of plasma and serum were stored at −80 ºC until analysis of sdLDL and thawed only once. Participants were divided into groups based on their age: <35 years old (young adults) and ≥35 years old (middle-aged and older adults). The selection of the cut-off value for the division into age groups was based on the results of The Framingham Heart Study, where women and men aged ≥35 years were assigned a positive scale point value, which, when analyzed independently of other factors, indicated 1.7 and 2.3% risk of CVD in the next 10 years, respectively [40].

### 4.3. Laboratory Methods

Fasting glucose concentration was determined in blood plasma, while lipid parameters—total cholesterol, high-density lipoproteins cholesterol, low-density lipoproteins cholesterol, and triglycerides—were determined in the blood serum. All biochemical parameters were measured on the day of sampling with the Konelab 20i Thermo Scientific autoanalyzer (Vantaa, Finland), and intra- and inter-assay coefficients of variation were as follows: for FG, 1.13% and 1.99%; for TC, 1.72% and 2.27%; for HDL-C, 1.33% and 2.42%; for LDL-C, 2.7% and 4.9%; for TG, 1.74% and 4.08%, respectively. Serum aliquots were also stored at −80 ºC until sdLDL subclass determination by the precipitation method as described in detail by Hirano et al. [41]. Briefly, the serum sample was mixed 1:1 with a reagent containing heparin and MgCl_2_, incubated at 37 °C, cooled, and centrifuged, and the concentration of LDL cholesterol in the obtained supernatant was measured using Konelab 20i Thermo Scientific autoanalyzer. The concentration of LDL in supernatant corresponded to the concentration of sdLDL fraction. 

### 4.4. Atherogenicity Indexes Calculation

The concentration of non-HDL-C was calculated by subtracting HDL-C from TC. The TC/HDL is determined as the ratio of TC to HDL-C [42], while API is the logarithm of the ratio of TG to HDL-C [43].

### 4.5. Diagnostic Criteria of Metabolic Disorders

In order to identify the metabolic disorders associated with an increased risk of cardiovascular diseases, the following diagnostic criteria were used: TC ≥ 5.0mmol/L; TG ≥ 1.7mmol/L; HDL women < 1.2mmol/L; HDL men <1.0mmol/L; LDL-C ≥ 3.0mmo/L; non-HDL ≥ 3.4mmo/L [1]; glucose ≥ 5.5mmol/L [44]; TC/HDL women ≥ 4.0; TC/HDL men ≥ 4.5 [16]; API ≥ 0.15 [45,46].

### 4.6. Statistical Analysis

For all analyzed quantitative parameters, the consistency of their distribution with normal distribution was checked using the Shapiro–Wilk test. Quantitative variables with the normal distribution of values are presented as mean and the standard deviation (mean ±SD), while with non-normal distributions, as median and 25–75% of observed values (Me [Q1–Q3]). Quality variables were presented as the number of observations with the percentage in the appropriate group (n (%)), and the chi2 test was used for comparison. Comparisons between qualitative data were performed using Student’s t-test or the U Man–Whitney test, depending on the fulfilment of the condition of normal distribution. The non-parametric Kruskal–Wallis test was used to compare the four groups representing the quartiles of sdLDL concentration according to their deviation from the normal distribution in the selected groups. For the correlation analysis, the variables with non-normal distribution were subjected to logarithmic transformation to bring their distribution closer to normal. As a result, all correlation analyses could be performed with parametric tests. Correlations between two variables were examined using the Pearson correlation test, while multiple forward regression was used to test linear relationships between more than two independent variables. For qualitative variables belonging to specific sdLDL quartiles and the values of atherogenicity indicators associated with an increased risk of CVD, a logistic regression analysis was used. Statistical analysis was performed with the use of Statistica 13.3 (Statsoft Inc., Tulsa, OK, USA), and the probability value (*p*) < 0.05 was assumed as significant for all the statistical tests used.

## 5. Conclusions

Regardless of the presence of other recognized risk factors, such as hypertension or obesity, the mere performance of basic laboratory tests of the blood lipid profile may indicate the presence of a factor directly responsible for the development of CVD, i.e., high sdLDL concentration. In our study, a high concentration of sdLDL was also observed in people under 35 years old and differences in the correlation of sdLDL levels with concentrations of other lipid parameters were observed in both distinguished age groups. High sdLDL concentrations are related to higher values of the traditional biochemical risk factors of CVD, except for the lower HDL concentration observed in women in both study groups. Using generally accepted cut-offs for standard risk factors for cardiovascular disease, the observed abnormal API values in young people and the abnormal non-HDL values in older subjects are associated with a significantly increased chance of a high concentration of atherogenic sdLDL. Further studies are needed to establish decision values for sdLDL, and thus determine the cut-off for the commonly measured lipid parameters, which will enable the patient to qualify for further specialist diagnostics concerning the risk of cardiovascular diseases.

Generally, the identification of abnormal values for standard lipid profile parameters and API should be a reason for the specialized examination of sdLDL. Confirmation of high sdLDL concentration in young people is an indication of the need for action to prevent the development of cardiovascular disease. Small dense LDL, as one of the most atherogenic molecules, can be used to monitor the progress of anti-atherogenic therapy. The introduction of sdLDL concentration measurement is possible and recommended for specialized laboratories.

### 5.1. The Strength of the Study

Our study is an important contribution to understanding the factors related to the formation of the most atherogenic sdLDL particles and their interrelationships with other lipid parameters. Despite the proven involvement of sdLDL in atherogenesis, there has been a lack of studies conducted among the youngest adults or, in particular, involving the simultaneous analysis of a homogeneous population over a wide age range in the central European region. The strength of the study was its inclusion of a group of participants who were not taking medications that directly affect lipid parameters and blood glucose concentration. In the present study, we confirmed the dependence between sdLDL concentration and the distinctness of lipid factors, and that the strength of this dependency is age-related. Our findings justify planning and conducting further cross-sectional and long-term multi-center observational studies to confirm the necessity of using sdLDL level determination in primary and secondary prevention of CVD, also among young adults. Developing research into new markers related to residual risk, independent of total LDL concentration, will significantly reduce the incidence of cardiovascular events, improve the quality of life of the population, and reduce the public cost of health care in this regard.

### 5.2. Limitations of the Study

The limitations of this study were the sample size and the not exactly equal proportion of men and women in each of the investigated groups of subjects, as well as the single-center nature of the study. Our study of sdLDL concentration was carried out by the precipitation method, which requires a stage of preliminary preparation of the serum sample. However, such a measurement system enables the implementation of the method in almost every diagnostic laboratory, which determines the basic parameters of the lipid profile, including LDL, by a direct method and with basic equipment. In contrast to the homogeneous and fully automated method, the precipitation method is cheaper but requires more manual work.

## Figures and Tables

**Table 1:** Baseline characteristics between studied age groups.

Parameter	Age <35 y. *N* = 279 Mean ± SD (Me [Q1–Q3])	Age ≥ 35 y. *N* = 206 Mean ± SD (Me [Q1–Q3])	*p*
Age [years]	25.0 [22.0–28.0]	51.0 [40.0–64.0]	<0.001
Women, n(%)	189 (67.7)	124 (60.2)	0.086
Current smoker, n(%)	50 (18%)	43 (21%)	0.502
FG, mmol/L	4.87 ± 0.48	5.33 ± 0.81	<0.001
TC, mmol/L	4.45 ± 0.49	5.33 ± 1.02	<0.001
HDL-C Women, mmol/L	1.51 ± 0.31	1.45 ± 0.31	0.140
HDL-C Men, mmol/L	1.28 ± 0.26	1.28 ± 0.33	0.928
LDL-C, mmol/L	2.52 ± 0.66	3.33 ± 0.85	<0.001
non-HDL-C, mmol/L	3.02 ± 0.77	3.94 ± 0.99	<0.001
TG, mmol/L	0.89 [0.66–1.24]	1.26 [0.96–1.77]	<0.001
TC/HDL Women	2.85 [2.54–3.23]	3.67 [3.17–4.31]	<0.001
TC/HDL Men	3.52 [3.15–3.98]	4.08 [3.40–4.93]	0.001
API Women	−0.240 ± 0.213	−0.065 ± 0.261	<0.001
API Men	−0.470 ± 0.253	0.027 ± 0.279	0.072
sdLDL, mmol/L	0.71 [0.46–1.03]	1.14 [0.62–1.70]	<0.001

FG—fasting glucose, TC—total cholesterol, HDL-C—High-Density Lipoprotein cholesterol, LDL-C—Low-Density Lipoprotein cholesterol, non-HDL—non-High-Density Lipoprotein cholesterol, TG—Triglycerides, TC/HDL—total cholesterol to High-Density Lipoprotein cholesterol ratio, API—Atherogenic Plasma Index, sdLDL—small, dense Low-Density Lipoprotein cholesterol.

**Table 2:** The results of Pearson correlation analysis for sdLDL with metabolic parameters and atherogenic indexes in both age groups.

Parameter	Age <35 y. *N* = 279 r; p	Age ≥35 y. *N* = 206 r; p	*p*^a^ Value for Correlation Coefficient Comparison
FG, mmol/L	−0.044; 0.462	0.096; 0.170	0.130
TC, mmol/L	0.197; 0.001	0.441; <0.001	0.003
HDL-C, mmol/L	−0.164; 0.006	0.054; 0.444	0.018
LDL-C, mmol/L	0.130; 0.030	0.380; <0.002	0.004
non-HDL-C, mmol/L	0.260; <0.001	0.442; <0.001	0.024
TG, mmol/L	0.321; <0.001	0.347; <0.001	0.752
TC/HDL	0.296; <0.001	0.282; <0.001	0.869
API	0.328; <0.001	0.245; <0.001	0.328

^a^ *p*-value for correlation coefficient comparison between age groups. FG—fasting glucose, TC—total cholesterol, HDL-C—high-density lipoprotein cholesterol, LDL-C—low-density lipoprotein cholesterol, non-HDL—non-high-density lipoprotein cholesterol, TG—triglycerides, TC/HDL—total cholesterol to high-density lipoprotein cholesterol ratio, API—atherogenic plasma index, sdLDL—small dense low-density lipoprotein cholesterol.

**Table 3:** Multiple forward stepwise regression analysis of sdLDL.

	Age < 35 Year		Age ≥ 35 Years	
	β Standardized; *p*	R^2^ for Model	β Standardized; *p*	R^2^ for Model
non-HDL	0.132; 0.043	0.121	0.358; <0.001	0.216
API	0.264; <0.001		-	
TG	-		0.168; 0.020	

Both models were adjusted by non-HDL cholesterol, triglycerides, TC/HDL, and API. *p*-value < 0.05 was considered significant. Non-HDL—non-high-density lipoprotein cholesterol, TG—triglycerides, API—atherogenic plasma index, sdLDL—small dense low-density lipoprotein cholesterol.

## Data Availability

Data available in a publicly accessible repository that does not issue DOIs. This data can be found here: [https://ppm.umed.wroc.pl/info/researchdata/UMW8e0ef68d50b24673aa2567b145ee351a/] accessed: 29 April 2022.

## Supplementary Materials

The following supporting information can be downloaded at: https://www.mdpi.com/article/10.3390/ijms23095089/s1.

## Abbreviations

API	atherogenic plasma index
CVD	cardiovascular disease
FG	fasting glucose
GLU	glucose
HDL-C	high-density lipoprotein cholesterol
LDL-C	low-density lipoproteins
non-HDL	non-high-dense lipoproteins
sdLDL	small dense low-density lipoproteins
TC	total cholesterol
TC/HDL	total cholesterol to high-dense lipoprotein ratio
TG	triglycerides
