# Supplementary material for: Atherogenic Plasma Index or Non-High-Density Lipoproteins as Markers Best Reflecting Age-Related High Concentrations of Small Dense Low-Density Lipoproteins
Source: Int J Mol Sci. 2022 May 3;23(9):5089. doi: 10.3390/ijms23095089 (PMC9102874; doi:10.3390/ijms23095089)
Supplement: Supplementary file 1 [file ijms-23-05089-s001.zip › Table S1.pdf]

Table S1. sdLDL Quartile characteristic among healthy participants

Parameter	sdLDL Quartile 1 <0.46 mmol/L N=36	sdLDL Quartile 2 ≥0.46 - <0.62 mmol/L N=31	sdLDL Quartile 3 ≥0.62 - <1.03 mmol/L N=46	sdLDL Quartile 4 >1.03 mmol/L N=39	*p* value for model
Women, n(%)	25 (69.4)	24 (77.4)	32 (69.6)	33 (84.6)	0.324
Age <35 y. n(%)	32 (94.1%)	29 (93.5%)	43 (95.6%)	33 (84.6%)	0.267
FG, mmol/L	4.76 [4.48-5.01]	4.82 [4.54-5.04]	4.70 [4.48-5.04]	4.70 [4.37-5.04]	0.865
TC, mmol/L	3.98 [3.75-4.44]	4.08 [3.82-4.42]	4.31 [4.08-4.50]	4.31 [4.08-4.52]	**0.035**[a]
HDL-C, mmol/L Women	1.58 [1.42-1.78]	1.60 [1.47-1.72]	1.60 [1.50-1.87]	1.47 [1.42-1.57]	0.087
HDL-C, mmol/L Men	1.24 [1.15-1.65]	1.24 [1.08-1.32]	1.29 [1.14-1.42]	1.21 [1.06-1.29]	0.685
LDL-C, mmol/L	2.09 [1.87-2.38]	2.22 [1.81-2.58]	2.35 [2.04-2.56]	2.30 [2.09-2.61]	0.107
non-HDL -C, mmol/L	2.51 [2.23-2.82]	2.79 [2.01-2.97]	2.76 [2.45-3.00]	2.84 [2.53-2.97]	0.067
TG, mmol/L	0.90 [061-1.09]	0.81 [0.56-1.00]	0.86 [0.60-1.04]	0.87 [0.74-1.37]	0.180

TC/HDL	2.71 [2.46-2.91]	2.76 [2.36-3.15]	2.77 [2.44-3.12]	2.87 [2.54-3.16]	0.391
API	-0.22 [-0.42-(-0.17)]	-0.26 [-0.52-(-0.13)]	-0.27 [-0.39-(-0.12)]	-0.20 [-0.30-(-0.03)]	0.210

[a] post-hoc test showed no significant differences

FG- Fasting Glucose, TC - Total Cholesterol, HDL-C - High Density Lipoprotein Cholesterol, LDL-C - Low Density Lipoprotein Cholesterol, non-HDL - non-High Density Lipoprotein Cholesterol, TG - Triglicerydes, TC/HDL - Total Cholesterol to High Density Lipoprotein Cholesterol Ratio, API - Atherogenic Plasma Index, sdLDL - small, dense Low Density Lipoprotein Cholesterol,
