# Supplementary material for: Atherogenic Plasma Index or Non-High-Density Lipoproteins as Markers Best Reflecting Age-Related High Concentrations of Small Dense Low-Density Lipoproteins
Source: Int J Mol Sci. 2022 May 3;23(9):5089. doi: 10.3390/ijms23095089 (PMC9102874; doi:10.3390/ijms23095089)
Supplement: Supplementary file 1 [file ijms-23-05089-s001.zip › Table S2.pdf]

Table S2. The frequency of metabolic disorders in all study participants, regarding to the age group

Parameter	Age <35 years N=279 W=189, M=90 n (%)	Age ≥35 years N=206 W=124, M=82 n (%)	*p*
FG, ≥5.5 mmol/L	31 (11.1)	59 (28.6)	**<0.001**
TC, ≥5.0 mmol/L	68 (24.3)	129 (62.6)	**<0.001**
HDL-C, ≤1.2 mmol/L Women	47 (24.9)	34 (27.4)	0.614
HDL-C, <1.0 mmol/L Men	11 (12.2)	16 (19.5)	0.189
LDL-C, ≥3.0 mmol/L	67 (24.0)	128 (62.1)	**<0.001**
non-HDL-C, ≥3.4 mmol/L	71 (25.4)	140 (68.0)	**<0.001**
TG, ≥1.7 mmol/L	27 (9.7)	58 (28.2)	**<0.001**
TC/HDL Women, ≥4.0	16 (8.5)	44 (35.5)	**<0.001**
TC/HDL Men,≥ 4.5	18 (20.0)	28 (34.1)	**0.036**
API,≥ 0.15	26 (9.3)	44 (21.4)	**<0.001**

W – women, M – Men, FG - Fasting Glucose, TC - Total Cholesterol, HDL-C - High Density Lipoprotein Cholesterol, LDL-C - Low Density Lipoprotein Cholesterol, non-HDL - non-High Density Lipoprotein Cholesterol, TG - Triglicerydes, TC/HDL - Total Cholesterol to High Density Lipoprotein Cholesterol Ratio, API - Atherogenic Plasma Index,
