# Supplementary material for: Atherogenic Plasma Index or Non-High-Density Lipoproteins as Markers Best Reflecting Age-Related High Concentrations of Small Dense Low-Density Lipoproteins
Source: Int J Mol Sci. 2022 May 3;23(9):5089. doi: 10.3390/ijms23095089 (PMC9102874; doi:10.3390/ijms23095089)
Supplement: Supplementary file 1 [file ijms-23-05089-s001.zip › Table S3.pdf]

Table S3. Small dense LDL Quartile characteristic among all study participants

Parameter	sdLDL Quartile 1<br><0.46 mmol/L<br>N=73<br>(Me [Q1-Q3])	sd LDL Quartile 2<br>≥0.46–<0.62 mmol/L<br>N=77<br>(Me [Q1-Q3])	sdLDL Quartile 3<br>≥0.62–<1.03 mmol/L<br>N=183<br>(Me [Q1-Q3])	sdLDL Quartile 4<br>>1.03 mmol/L<br>N=152<br>(Me [Q1-Q3])	*p*
Women, n(%)	52 (71.2)	46 (59.7)	115 (62.8)	100 (65.8)	0.468
Age <35 y. n(%)	53 (72.6)	45 (58.44)	100 (54.6)	81 (53.3)	**0.036**<br>***p* for trend = 0.012**
FG,mmol/L	4.76 [4.48-5.21]	5.04 [4.65-5.49]	4.98 [4.65-5.43]	5.04 [4.62-5.52]	0.057
TC,mmol/L	4.21 [3.80-4.65]	4.31 [3.82-4.73]	4.93 [4.41-5.50]<br>vs.Q1; p<0.001<br>vs.Q2; p<0.001	4.88 [4.30-4.93]<br>vs.Q1; p<0.001<br>vs.Q2; p<0.001	**<0.001**
HDL-C,mmol/L Women	1.38 [1.22-1.65]	1.47 [1.32-1.70]	1.50 [1.29-1.73]	1.44 [1.29-1.70]	0.395
HDL-C,mmol/L Men	1.34 [1.19-1.58]	1.24 [1.08-1.37]	1.33 [1.11-1.51]	1.12 [0.98-1.34]<br>vs.Q1; p=0.003<br>vs.Q3; p=0.005	**<0.001**

LDL-C,mmol/L	2.30 [2.07-2.69]	2.47 [2.09-2.89]	3.02 [2.43-3.49]<br>vs.Q1; p<0.001<br>vs.Q2; p<0.001	2.95 [2.30-3.86]<br>vs.Q1; p<0.001<br>vs.Q2; p<0.001	**<0.001**
non-HDL-C,mmol/L	2.76 [2.48-3.15]	2.92 [2.48-3.23]	3.51 [2.92-4.08]<br>vs.Q1; p<0.001<br>vs.Q2; p<0.001	3.51 [2.87-4.55]<br>vs.Q1; p<0.001<br>vs.Q2; p<0.001	**<0.001**
TG,mmol/L	0.89 [067-1.15]	0.84 [0.61-1.18]	1.06 [0.81-1.58]<br>vs. Q1; p<0.001<br>vs. Q2; p<0.001	1.24 [0.85-1.84]<br>vs. Q1; p<0.001<br>vs. Q2; p<0.001	**<0.001**
TC/HDL	2.94 [2.62-3.39]	3.15 [2.68-3.61]	3.52 [2.91-4.18]<br>vs. Q1; p<0.001<br>vs. Q2; p=0.023	3.59 [2.98-4.58]<br>vs. Q1; p<0.001<br>vs. Q2; p=0.001	**<0.001**
API	-0.21 [-0.33- (0.08)]	- 0.19 [-0.34-(-0.02)]	-0.12 [-0.29-0.07]<br>vs. Q1; p=0.038	-0.04 [-0.22-0.13]<br>vs. Q1; p<0.001<br>vs. Q2; p<0.001	**<0.001**

FG- Fasting Glucose, TC - Total Cholesterol, HDL-C - High Density Lipoprotein Cholesterol, LDL-C - Low Density Lipoprotein Cholesterol, non-HDL - non-High Density Lipoprotein Cholesterol, TG - Triglicerydes, TC/HDL - Total Cholesterol to High Density Lipoprotein Cholesterol Ratio, API - Atherogenic Plasma Index, sdLDL - small, dense Low Density Lipoprotein Cholesterol,
